# Supplementary material for: Examining the Effects of Temperature on the Evolution of Bacterial tRNA Pools
Source: Genome Biol Evol. 2024 May 28;16(6):evae116. doi: 10.1093/gbe/evae116 (PMC11166485; doi:10.1093/gbe/evae116)
Supplement: evae116_Supplementary_Data [file evae116_supplementary_data.docx]

**Supplemental Table**

| **Phylogenetic Regression** | **Model** | **AIC** | **∆AIC (relative to best)** |
| --- | --- | --- | --- |
| GC vs. tRNA Diversity | BM | 1796.698 | 0 |
|  | OU | 1797.452 | 0.754 |
| tRNA Diversity vs. missense error rate | BM | -5956.54 | 140.5 |
|  | OU | -6097.04 | 0 |
| GC vs. missense error rate | BM | -5948.735 | 118.309 |
|  | OU | -6067.044 | 0 |
| GC vs. tRNA Diversity (GC-ending codons) | BM | 1791.535 | 0 |
|  | OU | 1793.672 | 2.137 |
| GC vs. tRNA Diversity (AT-ending codons) | BM | 653.397 | 77.831 |
|  | OU | 575.566 | 0 |

Table S1. Model fit comparison of phylogenetic regressions based on either the Brownian Motion (BM) or Ornstein-Uhlenbeck (OU) models of trait evolution. Model comparisons are based on the Akaike Information Criterion (AIC). Models with the lower AIC are considered the better model.
